# Improving anti-tumor efficacy of low-dose Vincristine in rhabdomyosarcoma *via* the combination therapy with FOXM1 inhibitor RCM1

**DOI:** 10.3389/fonc.2023.1112859

**Published:** 2023-02-02

**Authors:** Johnny Donovan, Zicheng Deng, Fenghua Bian, Samriddhi Shukla, Jose Gomez-Arroyo, Donglu Shi, Vladimir V. Kalinichenko, Tanya V. Kalin

**Affiliations:** ^1^ Division of Pulmonary Biology, Cincinnati Children’s Hospital Medical Center, Cincinnati, OH, United States; ^2^ The Materials Science and Engineering Program, College of Engineering and Applied Science, University of Cincinnati, Cincinnati, OH, United States; ^3^ Center for Lung Regenerative Medicine, Cincinnati Children’s Hospital Medical Center, Cincinnati, OH, United States; ^4^ Division of Pulmonary and Critical Care and Sleep Medicine, Department of Internal Medicine, University of Cincinnati, Cincinnati, OH, United States

**Keywords:** FOXM1 inhibitor, combination therapy, rhabdomyosarcoma, nanoparticles, animal models

## Abstract

Rhabdomyosarcoma (RMS) is a highly metastatic soft-tissue sarcoma that often develops resistance to current therapies, including vincristine. Since the existing treatments have not significantly improved survival, there is a critical need for new therapeutic approaches for RMS patients. FOXM1, a known oncogene, is highly expressed in RMS, and is associated with the worst prognosis in RMS patients. In the present study, we found that the combination treatment with specific FOXM1 inhibitor RCM1 and low doses of vincristine is more effective in increasing apoptosis and decreasing RMS cell proliferation *in vitro* compared to single drugs alone. Since RCM1 is highly hydrophobic, we developed innovative nanoparticle delivery system containing poly-beta-amino-esters and folic acid (NP^FA^), which efficiently delivers RCM1 to mouse RMS tumors *in vivo*. The combination of low doses of vincristine together with intravenous administration of NP^FA^ nanoparticles containing RCM1 effectively reduced RMS tumor volumes, increased tumor cell death and decreased tumor cell proliferation in RMS tumors compared to RCM1 or vincristine alone. The combination therapy was non-toxic as demonstrated by liver metabolic panels using peripheral blood serum. Using RNA-seq of dissected RMS tumors, we identified *Chac1* as a uniquely downregulated gene after the combination treatment. Knockdown of *Chac1* in RMS cells *in vitro* recapitulated the effects of the combination therapy. Altogether, combination treatment with low doses of vincristine and nanoparticle delivery of FOXM1 inhibitor RCM1 in a pre-clinical model of RMS has superior anti-tumor effects and decreases CHAC1 while reducing vincristine toxicity.

## Introduction

Rhabdomyosarcoma (RMS) is the most common soft tissue sarcoma in children, accounting for about 350 cases in the United States each year (1–5). RMS is highly metastatic, and these patients have a poor prognosis, with the 5-year survival rate being around 20% (2–4). Chemotherapy is often used to treat these patients, such as anthracycline-based agents like doxorubicin or epirubicin, alkylating agents like ifosfamide and cyclophosphamide, cytotoxic therapeutics like dactinomycin, and vincristine (VCR), a vinca alkaloid agent (6). VCR is a cell-cycle specific therapeutic that has been widely accepted as a preferred initial, recurrent, and metastatic treatment for RMS patients (2, 7, 8). Unfortunately, many chemotherapies including VCR are toxic, leading to weight loss, decreased blood counts, and neuropathy (2, 5, 8, 9). VCR is used in combination with other therapeutics to combat toxicity, while also increasing efficacy (5, 7). Despite this, RMS is highly recurrent and will often develop therapeutic resistance to these drugs (2, 3), which indicates an urgent need for new therapies.

FOXM1, a well-known oncogene, is an important transcription factor that is crucial to many pathways and processes, most notably proliferation, mitosis, and cell survival (10–14). FOXM1 is overexpressed in many cancers, including RMS, and the high-levels of FOXM1 are clinically associated with a worse prognosis in RMS patients (15, 16). FOXM1 is primarily expressed during embryogenesis in the developing tissue, with minimal-to-no expression in normal healthy tissue, making it a very attractive therapeutic target during carcinogenesis (17–20). Previous studies have shown that the loss of FOXM1 in tumor cells results in decreased tumor cell proliferation, metastasis, angiogenesis, and increased tumor cell death (21–24). However, many of the targeted approaches to inhibit FOXM1 have largely been unsuccessful due to their lack of specificity and off-target effects, including toxicity (17, 23).

Recently, we have identified a small molecule inhibitor of FOXM1 (Robert Costa Memorial Drug-1, RCM1) that has shown to be an effective anti-tumor agent and is also non-toxic in pre-clinical mouse models (19, 25). RCM1 removes FOXM1 from the nucleus into the cytoplasm, leading to FOXM1 ubiquitination and proteasomal degradation (25). Treatment with RCM1 reduces tumor cell proliferation, migration and colony formation while also inducing apoptosis across several cancer types, including RMS (19). RCM1 treatment increases duration of mitosis in tumor cells (19), suggesting that the use of RCM1 can synergize with anti-mitotic chemotherapeutic agents to increase the efficacy of anti-cancer treatment. Furthermore, dual-treatment with RCM1 and anti-mitotic drugs provides an opportunity to decrease the drug concentrations and decrease side effects of anti-cancer therapy as a result. In the present studies, we investigated the effect of RCM1 in combination with low dose of VCR in RMS mouse models. Our results demonstrate a combinatorial effect between RCM1 and VCR to increase anti-tumor efficacy on RMS tumors, potentially suggesting a novel therapeutic option for RMS patients.

## Materials and methods

### Cell lines and reagents

Human RD (ATCC) is an embryonal RMS cell line derived from a 7-year-old Caucasian female previously treated with cyclophosphamide and radiation. RD is treatment refractory (26), has MYC amplification (27), mutations in NRAS and TP53 (28), and is sensitive to VCR (29). Rd76-9 is a murine derived embryonal RMS cell line, isolated from a methylcholanthrene-induced mouse RMS tumor in a female C57BL/6 mouse (30) and was provided by Dr. Tim Cripe (Nationwide Children’s Hospital, Columbus, OH). The small molecule compound RCM1 (2-[2-oxo-2-(thiophen-2-yl) ethyl]sulfanyl -4,6-di(thiophen-2-yl)pyridine-3-carbonitrile) was synthesized by Vitas-M Laboratory (95% purity). Vincristine Sulfate (VCR, NDC 61703-309-16) was purchased through in-house pharmacy at Cincinnati Children’s Hospital.

### Nanoparticle synthesis

Bisphenol A glycerolate diacrylate, 4,4’-Trimethylenedipiperidine, 6-amino-1-hexanol, Oleic acid, polyethylenimine (PEI, Mn ~600) poly(ethylene glycol) (PEG, Mn ~2000), and Folic acid (FA) were purchased from Sigma-Aldrich (St. Louis, MO, USA). Lecithin (from Soybean) was purchased from Tokyo Chemical Industry Co., Ltd. (TCI). DyLight 650 or 800 NHS ester, carbodiimide hydrochloride (EDC), N- Hydroxysuccinimide (NHS), SnakeSkin™ Dialysis Tubing, and 10K MWCO were purchased from ThermoFisher Scientific (Waltham, MA, USA).

The amphiphilic poly-beta amino ester (aPBAE) nanoparticle backbone was synthesized *via* a Michael Addition. Briefly, the Bisphenol A glycerolate diacrylate was first mixed with 4,4’-Trimethylenedipiperidine in DMSO at 50°C for 24 hours. The mixture was then added to 6-amino-1-hexanol with the temperature increased to 90°C and held for another 24 hours. The polymer backbone was capped by a FA modified PEI. The FA and PEG modifications were processed *via* EDC/NHS coupling, as described previously (31, 32). To encapsulate RCM1, nanoparticle components were mixed with RCM1 in DMSO, then moved to an aqueous condition to allow DMSO to diffuse and the nanoparticles to assemble for 4 hours, followed by dialysis for 48 hours to remove DMSO and impurities. UV/Vis spectroscopy has been widely used to determine drug loading capacity using various solvents, including DMSO (33–35), so RCM1-encapsulated nanoparticles were characterized by using UV/Vis spectroscopy to determine the amounts of RCM1 according to the standard curve for estimation of encapsulation concentration.

### Mouse models

C56Bl/6J mice were purchased from the Jackson laboratory. To generate the subcutaneous syngeneic murine model, 1x10^6^ Rd76-9 rhabdomyosarcoma cells were re-suspended in equal volumes of PBS : Matrigel (Corning) and were injected into the flanks of 8-12 weeks old C57Bl/6J mice. Animals were treated with VCR or saline (control) every 7 days *via* intraperitoneal injections. Animals were treated with RCM1 encapsulated nanoparticles or empty nanoparticles (control) every 48 hours intravenously *via* the eye vein. Tumors were measured using calipers, and volumes were calculated in cubic millimeters using $\frac{1}{2}(L\times (W^{2})),$ where *L* is the largest diameter and *W* is the diameter perpendicular to *L*. Serum was collected from animals treated with empty nanoparticles, treated with 0.25mg/kg VCR alone, 8µg RCM1 nanoparticles alone or treated with combination of 0.25mg/kg VCR and 8µg RCM1 nanoparticles. Each animal study had 3-7 mice per group.

### *In vitro* growth curve analysis

3x10^5^ Tumor cells per well were seeded in 6-well plates and allowed to grow for 24 hours or 48 hours. After 24 hours, tumor cells were then treated with RCM1, VCR or RCM1 + VCR for 24 hours. Trypan blue was used to exclude dead cells and viable cells were counted using a Hemocytometer. Experiments were performed in triplicate.

### Immunostaining

3x10^5^ Tumor cells per well were seeded on 24mm square cover slips in 6-well plates and allowed to grow for 24 hours. Tumor cells were then treated with RCM1, VCR or RCM1 + VCR for 24 hours. Tumor cells were then fixed and stained as previously described (36). To visualize the nucleus, Hoechst 33342 (ThermoFisher Scientific) was used as a counter stain. For quantification, 5 random fields were acquired per sample and quantified using ImageJ. To perform immunostaining of tumor tissue, paraffin embedded Rd76-9 subcutaneous tumor sections were stained as described previously (37). 5 Random fields per sample were acquired and quantified using ImageJ. Antibodies used for immunostaining were anti-Ki-67 (Invitrogen, MA5-14520), anti-phospho-histone H3 (Santa Cruz, sc-374669 (C-2)), anti-CD31 (R&D, AF3628), anti-Cleaved-Caspase 3 (R&D, MAB835), anti-CHAC1 (Novus Biologicals, OTI1E2).

### qRT-PCR and western blot

For stable knockdown of CHAC1, Rd76-9 cells were transduced with GIPZ *Chac1* shRNA (V3LMM_499697, Horizon). GIPZ non-silencing lentiviral shRNA (RHS4346, Horizon) was used as control. Lysis Buffer (Qiagen) and β-Mercaptoethanol were used to lyse cells *in vitro*. Taqman gene expression assays for *Chac1* and *Beta-Actin* were purchased from ThermoFisher. qRT-PCR was performed as previously described (38, 39). Protein extracts were prepared as described (40, 41). Antibodies used for western blots were anti-CHAC1 (Sigma, AV42623), and anti-Vinculin (Cell Signaling, E1E9V).

### RNA-seq and data processing

Dissected Rd76-9 tumors from control (treated with empty nanoparticle and saline), 0.25mg/kg VCR, 8µg RCM1 and 0.25mg/kg VCR + 8µg RCM1 groups were snap frozen in liquid nitrogen 16 days post tumor inoculation. RNA was extracted from bulk tumor samples and were sent for sequencing. Quality of RNA was determined using Fragment Analyzer with an average RQN for all samples of 9.7. RNA libraries were prepared for all samples using Illumina TruSeq Stranded mRNA Prep to generate poly-A enriched, non-stranded RNA libraries. Sequencing was performed using NoveSeq6000 with an estimated 30 million read per sample. Reads were aligned to the GRCm38 mouse genome and quantified using an index transcriptome version of GRCm38 using *Kallisto* using standard settings (42). Raw counts were normalized using *DESeq2* (43). Differential gene expression between conditions was performed using DEseq2 which uses a negative binomial model for each gene. The Wald test was used for hypothesis testing when comparing the two groups. All p-values attained were corrected for multiple testing using the Benjamini and Hochberg method which is the default method in DEseq2. In the standard DESeq2 algorithm, *alpha* for false-discovery rate is set to 0.1 by default. Heatmap was generated using the *pheatmap* R package and the volcano plot was generated using the *EnhancedVolcano* R package (https://github.com/kevinblighe/EnhancedVolcano). Venn diagrams for differentially expressed genes were created using AltAnalyze (44).

### Statistical analysis

The Student *t* test, one-way ANOVA, one-way ANOVA RM and two-way ANOVA RM were used to determine statistical significance. *P*-values <0.05 were considered significant. Values were shown as mean SD. All statistical analyses were obtained using GraphPad Prism.

### Study approval

All animal studies were approved by Cincinnati Children’s Research Foundation Institutional Animal Care and Use Committee and covered under our animal protocol (IACUC2022-0041). The Cincinnati Children’s Research Foundation Institutional Animal Care and Use Committee is an AAALAC and NIH accredited institution (NIH Insurance #8310801).

## Results

### VCR and RCM1 synergize in murine RMS cells *in vitro*

**Figure 1:**
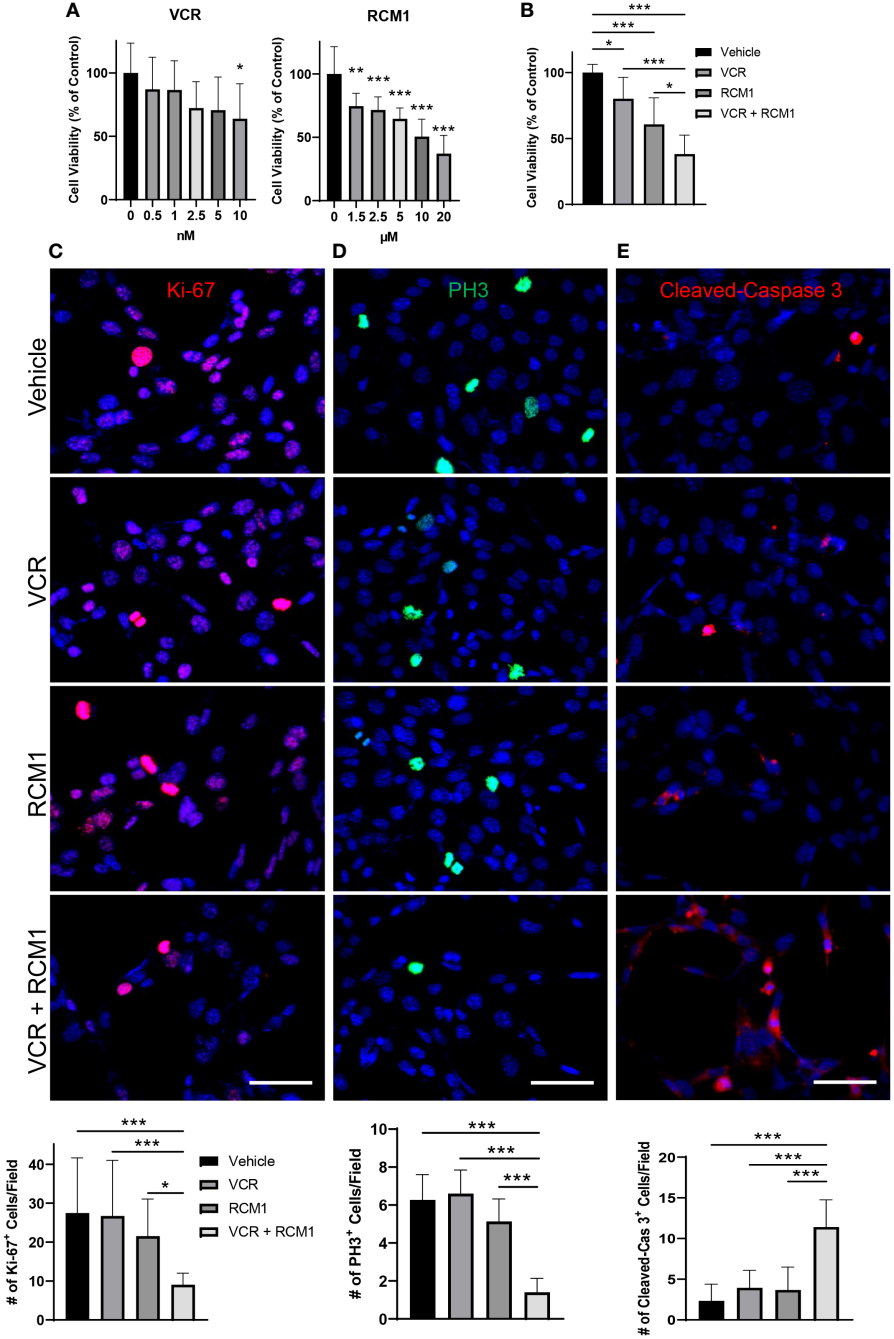
Combination of VCR and RCM1 is more efficient than single agents in reducing growth of murine Rd76-9 rhabdomyosarcoma cells *in vitro.* **(A)**, Left: Vincristine (VCR) reduced Rd76-9 tumor cell viability in a dose-dependent manner after 24 hours of treatment compared to vehicle (saline) control. Right: RCM1 reduced Rd76-9 tumor cell viability in a dose-dependent manner after 24 hours of treatment compared to vehicle (DMSO) control. Graphs show the percentage of live tumor cells per group compared to vehicle control. **(B)**, Combination therapy using IC_50_ concentrations of VCR (2.1nM) and RCM1 (8.7µM) reduced Rd76-9 tumor cell viability compared to single agents at IC_50_ concentrations and to vehicle (saline + DMSO). The graph shows the percentage of live tumor cells per group compared to vehicle control after 24 hours of treatment. **(C)**, Combination therapy decreased Rd76-9 tumor cell proliferation (Ki-67, red) and mitosis (**D**, PH3, green), and increased apoptosis (**E**, Cleaved-Caspase 3, red) compared to single agents or vehicle control after 24 hours of treatment. 5 random fields per sample were used to quantify the number of Ki-67^+^, PH3^+^ and Cleaved-Caspase 3^+^ cells per group. Values are shown as mean ± SD. *P<0.05; **P<0.01; ***P<0.001. Scale bar=50µm.

To determine the half maximal inhibitory concentrations (IC_50_) of VCR and RCM1, dose response curves were generated for each single agent. After 24 hours, both VCR and RCM1 reduced the number of Rd76-9 murine RMS cells in a dose-dependent manner (**Figure 1A**). Using the dose response curves for VCR and RCM1, we selected IC_50_ concentrations of 2.1nM for VCR and 8.7µM for RCM1 to use in combination treatment. Using these concentrations, the dual treatment with low doses of VCR and RCM1 synergized to reduce the number of Rd76-9 tumor cells after 24 hours compared to the single agents and control (**Figure 1B**, **S1A**). To further characterize this novel dual-drug therapy, we performed immunostaining of tumor cells using various markers. Since both RCM1 and VCR are cell-cycle specific anti-tumor agents, we first explored the effects of the combination therapy on tumor cell proliferation. Cell proliferation was assessed using immunostaining for Ki-67, a general proliferation marker, and phospho-histone H3 (PH3), a mitosis specific marker. After 24 hours, the combination therapy synergized to reduce the number of Ki-67-positive cells compared to each single agent (**Figure 1C**). Also, the two agents synergized to reduce the number of mitotic cells after 24 hours (**Figure 1D**). To characterize the effects of this combination therapy on cell death, we performed immunostaining for Cleaved-Caspase 3, a well-known marker for apoptosis. After 24 hours, this combination therapy synergized to increase the number of apoptotic RMS cells, suggesting that the dual-drug therapy with low doses of RCM1 and VCR induces tumor cell death with higher efficiency than single agents alone (**Figure 1E**). These results demonstrate that RCM1 in combination with VCR synergize to inhibit proliferation and increase apoptosis of murine RMS tumor cells *in vitro*.

### VCR and RCM1 synergize in human RMS cells *in vitro*

**Figure 2:**
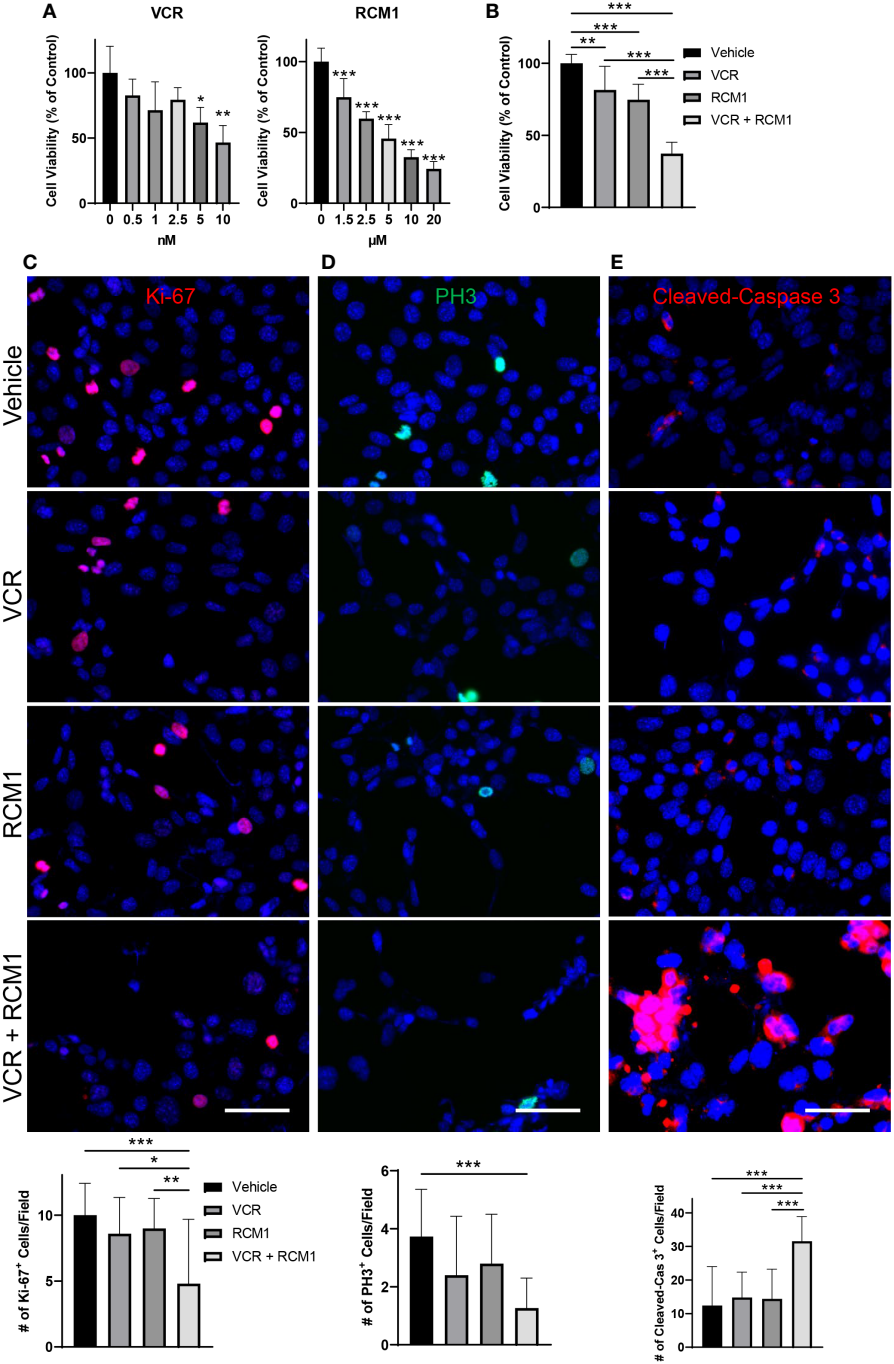
The combination of VCR and RCM1 is more efficient than single agents in reducing growth of human RD rhabdomyosarcoma cells *in vitro.* **(A)**, Left: VCR reduces RD tumor cell viability in a dose-dependent manner after 24 hours of treatment compared to vehicle (saline) control. Right: RCM1 reduces RD tumor cell viability in a dose-dependent manner after 24 hours of treatment compared to vehicle (DMSO) control. Graphs show the percentage of live cells per group compared to vehicle control. **(B)**, Combination therapy using IC_50_ concentrations of VCR (3.5nM) and RCM1 (3.0µM) reduced tumor cell viability compared to single drugs or vehicle (saline + DMSO) control after 24 hours of treatment. The graph shows the percentage of live cells per group compared to vehicle control. **(C)**, Combination therapy reduces proliferation (Ki-67, red) of RD tumor cells compared to single drugs or vehicle control after 24 hours of treatment. **(D)**, Combination therapy reduces mitosis (PH3, green) of RD tumor cells compared to vehicle control after 24 hours of treatment. **(E)**, Combination therapy induces apoptosis (Cleaved-Caspase 3, red) of RD tumor cells compared to single drugs alone or vehicle control after 24 hours of treatment. 5 random fields per sample were used to quantify the number of Ki-67^+^, PH3^+^ and Cleaved-Caspase 3^+^ cells per group. Values are shown as mean ± SD. *P<0.05; **P<0.01; ***P<0.001. Scale bar=50µm.

Since the dual treatment with low doses of RCM1 and VCR was effective in murine RMS cells, we investigated the effects of this combination therapy using a human derived RMS cell line, RD. To determine an optimal treatment dose, we generated the dose response curves for each drug and selected 3.5nM for VCR and 3.0µM for RCM1 to use in combination therapy (**Figure 2A**). Consistent with the mouse data, this dual therapy with low doses of VCR and RCM1 synergized to reduce the number of human RD tumor cells more efficiently compared to single agents (**Figure 2B**, **S1B**). Next, we assessed tumor cell proliferation by performing immunostaining for Ki-67 and PH3. Similar to the murine RMS cells, the combination therapy synergized to reduce the number of Ki-67-positive proliferating tumor cells (**Figure 2C**) and PH3-positive mitotic tumor cells compared to single agents (**Figure 2D**). Importantly, combination of low doses of VCR and RCM1 synergized to increase the number of apoptotic tumor cells as shown by immunostaining for Cleaved-Caspase 3 (**Figure 2E**). Taken together, these data suggest that the RCM1 and VCR combination therapy can be a promising effective therapy for RMS *in vivo*.

### Treatment with either VCR or RCM1 alone reduces tumor burden in a mouse model of RMS

To determine the half maximal inhibitory concentrations (IC_50_) of VCR and RCM1 as single agents *in vivo*, Rd76-9 murine RMS cells were injected subcutaneously into the flanks of C57Bl/6J mice to create a syngeneic RMS model. Mice were treated with VCR at days 7 and 14 after tumor cell inoculation (**Supplementary Figure S2A**). Treatment with VCR reduced tumor volume in a dose-dependent manner (**Supplementary Figure S2B**). Using tumor volumes measured at day 16, we generated a dose response curve to determine the IC_50_ concentration for VCR (0.25mg/kg) that later would be used for the combination therapy with RCM1 (**Supplementary Figure S2C**). Since VCR can be toxic (5, 8, 9), the animals were weighed at harvest to compare body weights. There were no differences in body weights between groups, suggesting that these doses of VCR are non-toxic (**Supplementary Figure S2D**). Treatment with VCR reduced tumor cell proliferation and mitosis in a dose-dependent manner as demonstrated by immunostaining of RMS tumor sections for Ki-67 and PH3 (**Supplementary Figures S2E, F**). We next analyzed the tumor-associated angiogenesis using immunostaining for CD31, a marker of endothelial cells. There was no difference in endothelial coverage between VCR treated and control groups (**Supplementary Figure S2G**). The effect of VCR on apoptosis of RMS cells was not significant as shown by immunostaining for Cleaved-Caspase 3 (**Supplementary Figure S2H**).

**Figure 3:**
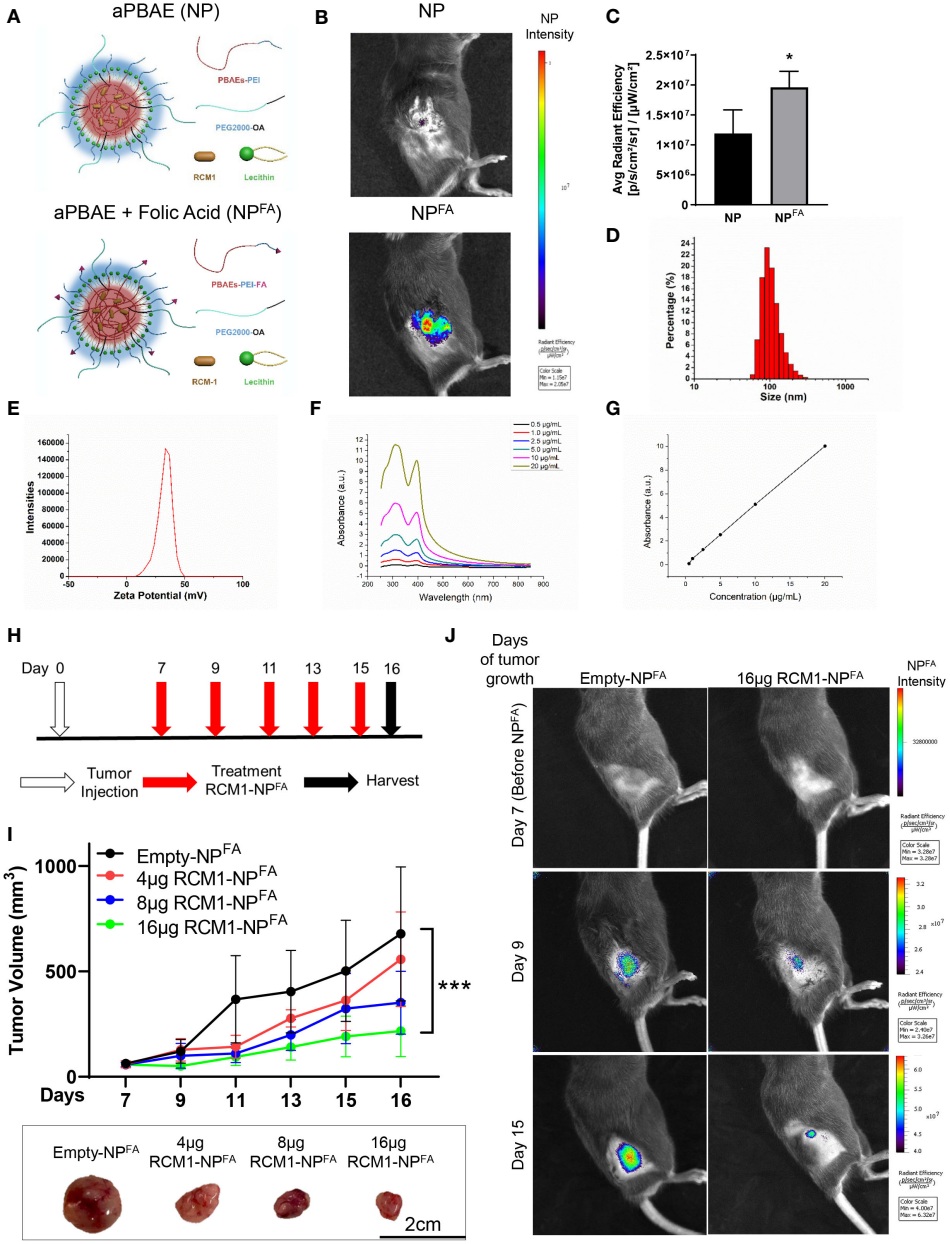
Generation of nanoparticle to deliver RCM1 to tumors. **(A)**, Graphic of amphiphilic poly-beta amino ester (aPBAE) nanoparticles without folic acid (NP, top) and with folic acid (NP^FA^, bottom). **(B)**, Highly efficient delivery of NP^FA^, compared to NP into the tumors is shown using IVIS imaging. Mice bearing Rd76-9 subcutaneous RMS tumors were injected with NP or NP^FA^ labeled with DyLight 800. NP^FA^ are present in the tumor 48 hours after i.v. injection. **(C)**, Average radiant efficiency indicating NP^FA^ have a higher intensity compared to NP. **(D)**, The sizes of NP^FA^ were measured and the hydrodynamic average diameter of NP^FA^ is 160.67nm. **(E)**, The surface charge of NP^FA^ is 38.13mV. **(F)**, The UV/Vis spectrum for RCM1 at increasing concentrations in DMSO determined RCM1 has an absorbance peak at 310nm and 395nm. **(G)**, Data from the UV/VIS spectra was used to generate a standard concentration curve that was used to determine RCM1 concentration in the nanoparticles. Adjusted R^2 =^ 0.99944. **(H)**, Schematic diagram showing treatment strategy of tumor bearing mice. Rhabdomyosarcoma Rd76-9 cells were inoculated subcutaneously. Animals were treated with 4µg, 8µg, or 16µg RCM1- NP^FA^. **(I)**, Treatment with RCM1-NP^FA^ reduced tumor burden in a dose-dependent manner in animals. Mice were treated with 4µg, 8µg, or 16µg of RCM1-NP^FA^ or with empty-NP^FA^. Tumor volumes were measured at different time points compared to empty-NP^FA^ (top panel). Representative tumors per group are shown (bottom panel). Values are shown as mean ± SD. n=3-7, *P<0.05; ***P<0.001. **(J)**, Presence of Empty-NP^FA^ and RCM1-NP^FA^ nanoparticles in the tumors are shown at days 9 and 15 after treatment. Nanoparticles were labeled with DyLight 800 and visualized using IVIS.

Even though RCM1 has been shown previously to be a promising anti-tumor agent (19), the pre-clinical and clinical delivery of RCM1 has some limitations since RCM1 is a highly hydrophobic compound. Nanoparticles have been shown to encapsulate highly hydrophobic anti-tumor agents and are used as a vehicle for drug delivery in patients (45–49). Since nanoparticles can be administered intravenously (i.v.), we tested whether hydrophobic RCM1 compound can be delivered to RMS tumors using nanoparticles. An amphiphilic poly-beta amino ester (aPBAE) backbone was used to generate nanoparticles. The amphiphilic polymers can self-assemble with cargo in an aqueous condition to form a hydrophobic core and a hydrophilic surface, which can keep the nanoparticle stable (50, 51). Next, lecithin was added to improve nanoparticle stabilization with PEG ligands (52, 53) (**Figure 3A**, upper panel). Finally, the folic acid molecules were incorporated into nanoparticles to increase their specificity towards tumor cells that overexpressed folate receptor (31, 54, 55) (**Figure 3A**, bottom panel). Nanoparticles were labeled with DyLight 800 to visualize their recruitment into RMS tumors in mice using IVIS. Compared to nanoparticles without folic acid (NP), the nanoparticles with folic acid (NP^FA^) demonstrated the most efficient localization to the RMS tumors (**Figure 3B**), with higher average radiant efficiency (**Figure 3C**). Based on high tumor specificity, we used the NP^FA^ nanoparticles thereafter. The sizes of NP^FA^ were measured and used to calculate the hydrodynamic average diameter of NP^FA^ as being 160.67nm (**Figure 3D**). The surface charge of NP^FA^ was 38.13mV (**Figure 3E**), which is a suitable surface charge for tumor cell targeting (32, 56, 57). Next, RCM1 was encapsulated into NP^FA^ nanoparticles. The UV/Vis spectrophotometry was used to determine the presence of RCM1 in the nanoparticles based on the RCM1 absorbance at 310nm and 395nm (**Figure 3F**). These UV/Vis spectra were used to generate a standard concentration curve and to calculate the amount of RCM1 encapsulated in the nanoparticles (**Figure 3G**). To test the anti-tumor efficacy of RCM1-NP^FA^, the tumor-bearing mice were treated with either control Empty-NP^FA^ or RCM1-NP^FA^ delivered i.v. every other day for 5 treatments (**Figure 3H**). Compared to Empty-NP^FA^, RCM1-containing nanoparticles reduced tumor volume in a dose-dependent manner (**Figures 3I, J**). A dose response curve was generated to determine the IC_50_ concentration for RCM1 in the nanoparticles, which was 8µg per injection (**Supplementary Figure S3A**). The animals were weighed at harvest. No differences in the body weights were found between mice treated with RCM1-NP^FA^ and control Empty-NP^FA^ (**Supplementary Figure S3B**). Treatment with RCM1-NP^FA^ reduced tumor cell proliferation in a dose-dependent manner as shown by immunostaining for Ki-67 and PH3 (**Supplementary Figures S3C, D**). Unlike treatment with VCR *in vivo*, the highest dose of RCM1-NP^FA^ reduced tumor-associated angiogenesis which was quantified as CD31^+^-vessel coverage within RMS tumors in mice (**Supplementary Figure S3E**). RCM1-NP^FA^ treatment did not induce tumor cell apoptosis at any concentration used (**Supplementary Figure S3F**). Collectively, these data demonstrate that both VCR and RCM1-NP^FA^ reduce tumor burden as single agents.

### Combination treatment of VCR and RCM1-NP^FA^ is more effective in reducing RMS tumor burden compared to single agents

**Figure 4:**
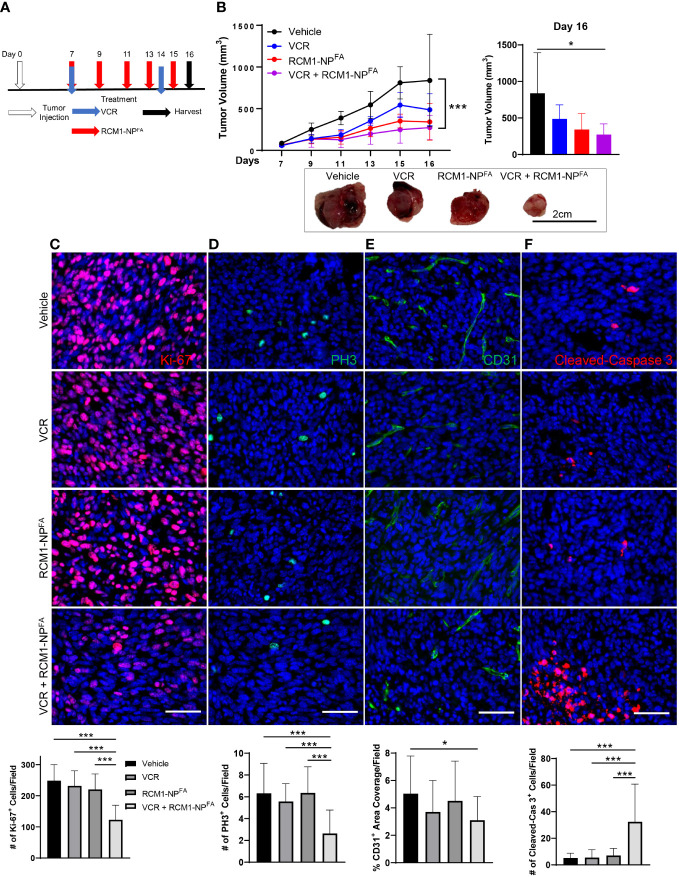
Combination treatment with low dose of VCR and RCM1-NP^FA^ is more efficient than single agents in reducing rhabdomyosarcoma tumor burden in mice. **(A)**, Experimental treatment strategy using low doses of VCR and RCM1-NP^FA^ as single agents or in combination. Mouse rhabdomyosarcoma Rd76-9 cells were subcutaneously injected. Tumor-bearing mice were treated with IC_50_ dose of VCR (0.25mg/kg), IC_50_ dose of RCM1-NP^FA^ (8µg) as single agents or in combination starting at day 7 after tumor cell inoculation. **(B)**, Combination therapy reduced tumor volume compared to vehicle (saline + empty-NP^FA^) control. Tumor volume measurements are shown at different time points (top left) and the end of the study (day 16, top right). Representative tumors per group are shown (bottom). **(C)**, Combination therapy is more efficient in reducing tumor cell proliferation (Ki-67, red) and mitosis (**D**, PH3, green) compared to single agents alone and vehicle control. **(E)**, Combination therapy reduces tumor-associated angiogenesis (CD31, green) compared to vehicle control. **(F)**, Combination therapy induces apoptosis (Cleaved-Caspase 3, red) compared to single agents and vehicle control. 5 random fields per sample were used to quantify the number of Ki-67^+^, PH3^+^, CD31^+^ and Cleaved-Caspase 3^+^ cells per group. Values are shown as mean ± SD. n=5-6, *P<0.05; ***P<0.001. Scale bar=50µm.

Using the IC_50_ concentrations determined for both VCR (0.25mg/kg) and RCM1 (8µg/injection) *in vivo* (**Supplementary Figure S2C** and **Supplementary Figure S3A** respectively), we next investigated the combinatorial effects of these drugs. The Rd76-9 RMS cells were inoculated subcutaneously into the flanks of mice and allowed tumor to grow for 7 days. On day 7, the tumor-bearing mice were treated with the first dose of both VCR and RCM1. VCR was administered once a week thereafter, and RCM1-NP^FA^ were administered every other day (**Figure 4A**). The combination of both drugs had shown the best efficacy in reducing tumor volumes (**Figure 4B**). There were no differences in body weights between control and all experimental groups of tumor-bearing mice (**Supplementary Figure S4A**). Blood was collected and a liver metabolic panel was analyzed from the serum of these mice. No differences were seen in the concentrations of albumin, total protein, ALP, AST, ALT, GGT and bilirubin (**Supplementary Figures S4B-D**). Taken together with the body weights, these results suggest that the combination therapy is non-toxic.

To characterize the effects of combination therapy on RMS tumors, the tumor sections were immunostained for Ki-67, PH3, CD31 and Cleaved-Caspase 3. Consistent with the *in vitro* data, the combination therapy synergized to reduce the number of proliferating cells in RMS tumors (**Figures 4C, D**). The combination therapy also reduced tumor-associated angiogenesis (**Figure 4E**). When assessing the cell death through Cleaved-Caspase 3 staining, we found that neither single agent at IC_50_ concentrations were able to increase tumor cell death. However, the combination therapy with non-toxic doses of VCR and RCM1-NP^FA^ synergized to increase tumor cell apoptosis (**Figure 4F**). Our results suggest that the nanoparticle delivery of RCM1 in combination with the low doses of VCR synergizes to improve efficacy of both drugs.

### The combination therapy induces a unique gene signature in RMS tumors

**Figure 5:**
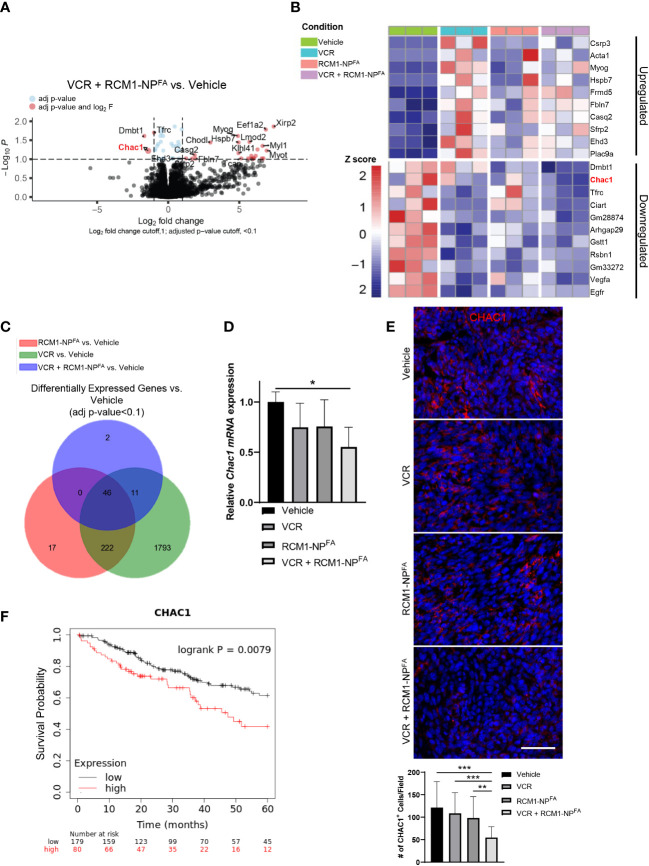
Treatment of mice with combination of low doses VCR and RCM1-NP^FA^ results in unique gene signatures. **(A)**, Volcano plot shows the differential gene expression between combo-treated (VCR + RCM1-NP^FA^) versus vehicle-treated (saline + empty-NP^FA^) tumors. RMS tumors were dissected from mice at day 16 after treatment with VCR, RCM1-NP^FA^ alone or in combination. Genes in blue were statistically significant with an adjusted p-value <0.1. Genes in red were statistically significant with a log_2_ fold-change higher than 1. **(B)**, Heat map summarizing centralized and scaled normalized counts (z-score) for the top 10 upregulated and downregulated genes in VCR + RCM1-NP^FA^ compared to vehicle control. **(C)**, Venn diagram showing the number of differentially expressed genes from tumors treated with VCR, RCM1-NP^FA^, and VCR + RCM1-NP^FA^ compared to vehicle control. **(D)**, *Chac1* is downregulated in the combination treated group compared to vehicle control. Bars represent fold changes compared to vehicle control using *beta-actin* as an internal control. Values are shown as mean ± SD. n=3 per group. *, *P*<0.05. **(E)**, Number of CHAC1^+^ cells (red) is reduced in combination therapy compared to each single agent alone and vehicle control *in vivo*. 5 random fields per sample were collected to quantify the number of CHAC1^+^ cells. Values are shown as mean ± SD. n=5-6, **P<0.01; ***P<0.001. **(F)**, Kaplan Meyer curves demonstrating that sarcomas expressing CHAC1 above the median value (high expression) had significantly worse outcomes compared to patients with sarcomas expressing CHAC1 below the median expression value (low expression), n=259.

To determine the molecular mechanism of synergistic effect after dual treatment with RCM1-NP^FA^ and VCR, the RNA-seq was performed to compare gene signatures of RMS tumors dissected from experimental and control groups of mice. RNA-seq analysis identified 59 differentially expressed genes in combination treated mice compared to vehicle (**Figure 5A**). Of which, ChaC Glutathione Specific Gamma-Glutamylcyclotransferase 1 (*Chac1*) was identified as one of the most downregulated genes in combination treated rhabdomyosarcoma tumors compared to vehicle-treated tumors (**Figures 5A, B**). Additionally, out of the 59 total differentially expressed genes in combination treated tumors, *Chac1* was one of the two genes that is uniquely downregulated only in combination treated tumors, but not in either single drug treated tumors (**Figure 5C**). To verify the RNAseq data, we performed qRT-PCR using bulk RNA from dissected tumors. *Chac1* mRNA was significantly decreased only in the tumors treated with combination therapy (**Figure 5D**). To further support our RNA-seq data, we examined the protein levels of CHAC1 after combination therapy in RMS tumors *in vivo*. Consistent with our RNA-seq and qRT-PCR data, the number of tumor cells expressing CHAC1 was reduced only after VCR+RCM1-NP^FA^ combination treatment, but not after either single agent (**Figure 5E**). Using TCGA datasets, we have also demonstrated that the high expression of CHAC1 was associated with a worse prognosis in sarcoma patients (**Figure 5F**), suggesting that CHAC1 can play a role in RMS tumorigenesis.

### Knockdown of CHAC1 leads to similar effects as combination therapy

**Figure 6:**
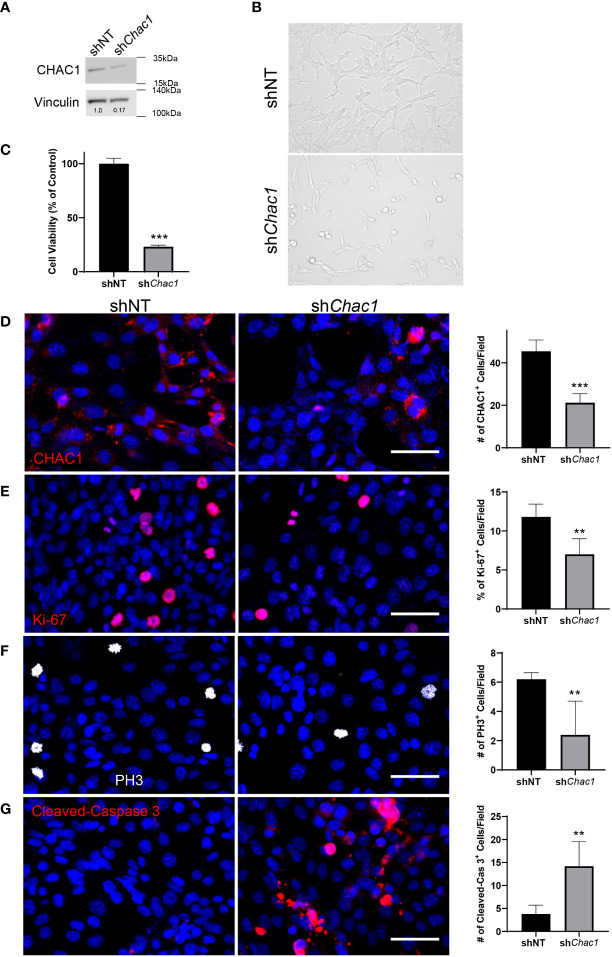
Depletion of *Chac1* decreases rhabdomyosarcoma tumor cell growth. **(A)**, Knockdown of *Chac1* in Rd76-9 RMS cells using sh*Chac1* efficiently decreases CHAC1 protein levels compared to shNT control, shown using Western blot. **(B)**, Bright field images of CHAC1-deficient Rd76-9 tumor cells show increased number of floating cells and decreased number of attached cells compared to control at 24 hours after seeding. **(C)**, Depletion of CHAC1 reduces the percent of vialble tumor cells compared to control at 48 hours after seeding. The graph shows the percentage of live cells compared to shNT control. **(D)**, Knockdown of *Chac1* in Rd76-9 RMS cells reduces the number of CHAC1^+^ (red) cells compared to shNT control. **(E)**, Knockdown of *Chac1* in Rd76-9 RMS cells reduces proliferation (Ki-67, red) and mitosis (**F**, PH3, white) compared to shNT. **(G)**, Knockdown of *Chac1* induces apoptosis in Rd76-9 tumor cells (Cleaved-Caspase 3, red) compared to shNT control. 5 random fields per sample were used to quantify the numbers of CHAC1^+^, Ki-67^+^, PH3^+^ and Cleaved-Caspase 3^+^ cells per group. Values are shown as mean ± SD. **P<0.01; ***P<0.001. Scale bar=50µm.

To determine the role of CHAC1 in RMS tumor cells, we generated a shRNA lentivirus against *Chac1* (sh*Chac1*) and transduced Rd76-9 cells to inhibit *Chac1* expression *in vitro*. CHAC1 protein levels in sh*Chac1* RMS tumor cells were decreased compared to non-targeting control, confirming efficient *Chac1* depletion (**Figure 6A**). Depletion of CHAC1 decreased the numbers of viable Rd76-9 tumor cells in culture compared to control tumor cells (**Figures 6B, C**). Immunostaining of Rd76-9 cells for CHAC1, Ki-67, PH3 and Cleaved-Caspase 3 protein demonstrated that the depletion of CHAC1 decreased the numbers of Ki-67^+^ and PH3^+^ proliferating tumor cells (**Figures 6D-F**) as well as increased the number of tumor cells undergoing apoptosis (**Figure 6G**). Taken together, the knockdown of CHAC1 in RMS tumor cells decreased cell proliferation and increased apoptosis, recapitulating the effects of the combination therapy with VCR and RCM1.

## Discussion

Rhabdomyosarcoma (RMS) is a highly aggressive pediatric soft tissue cancer with a poor prognosis (2–4). Vincristine (VCR) is a cell-cycle specific chemotherapeutic that is a first line of defense for these patients (7). However, VCR can be toxic (5), and many patients relapse or develop therapeutic resistance (2, 3), demonstrating an urgent need for new treatment strategies.

FOXM1 is an oncogene that is overexpressed in many cancers and has been clinically associated with a worse prognosis (15, 19). Despite the well-known roles of FOXM1 in cancer, there has been a lack of investigation of FOXM1 in RMS. FOXM1 is primarily expressed during embryogenesis, with minimal-to-no post-natal expression in normal healthy tissue, making it a very attractive therapeutic target (18, 19). There are many reports of pharmacological inhibition of FOXM1 (23, 24, 58). However, many of these drugs lack specificity, creating off-target effects, side effects, and are toxic (17, 23, 58). To date, there are not any clinically approved drugs to target FOXM1. We recently identified a small molecule inhibitor of FOXM1, Robert Costa Memorial Drug-1 (RCM1) that decreased tumorigenesis across multiple cancer types, including RMS, and is also non-toxic (19). FOXM1 is important in the cell-cycle, and interestingly, treatment with RCM1 increases the duration of the cell-cycle, suggesting that RCM1 can be used with other cell-cycle specific anticancer therapies for combinatorial effects.

In the present study, we demonstrated that low-dose concentrations of VCR and RCM1 in combination synergized to reduce tumor cells proliferation and mitosis across mouse and human RMS cell lines compared to each single agent. Remarkably, while neither low-dose concentration of VCR nor RCM1 could induce apoptosis alone, the combination therapy synergized to induce apoptosis of tumor cells *in vitro*.

To maintain clinical relevance, we generated VCR dose response curves *in vivo* using doses near the animal equivalency dose for patients (59). In addition, we administered VCR every 7 days, consistent with clinical use. As expected, the tumor burden and proliferation were reduced in a dose-dependent manner. Interestingly, these doses of VCR did not induce apoptosis, which could be explained due to the fact that the dosage used was low to avoid toxicity.

While RCM1 has been shown to be a promising anticancer therapy (19), there has been limitations to administration due to the high hydrophobicity of the drug. Previous reports have only been able to dissolve RCM1 in either pure DMSO, or a DMSO mixed solution, which is not a clinically relevant vehicle (19, 25). To make RCM1 administration more clinically relevant, we synthesized amphiphilic poly-beta amino ester (aPBAE) nanoparticles to use as a vehicle for RCM1 delivery. Nanoparticles have been used as a vehicle in the clinic for hydrophobic drugs like paclitaxel, as well as other RMS therapeutics like doxorubicin, and can be administered intravenously for several cancer types (60, 61). The aPBAE is one of the polymers that is used in generating nanoparticles and has recently attracted enormous attention in gene or drug delivery due to its excellent biocompatibility and biodegradability (62). The published studies combined aPBAEs and lipid molecules, including lecithin, to improve stability of nanoparticles (63). To further improve nanoparticle stabilization, lecithin was combined with PEG ligands, especially in liposomes or polymer-lipid hybrid nanoparticles (52, 53). To help guide the nanoparticles to the tumor, the newly synthesized aPBAE nanoparticles are also combined with folic acid, a metabolite that is readily up taken by tumors, due to the over-expression of folate acid receptors on the surfaces of tumor cells (31, 64). We demonstrated that RCM1-encapsulated nanoparticles can be injected intravenously and localize at the site of the tumor. Considering FOXM1 has minimal-to-no expression in normal healthy tissue, we did not expect any off-target effects. Mice treated with RCM1-encapsulated nanoparticles did not show any differences in body weights, suggesting RCM1-encapsulated nanoparticles are non-toxic, which is consistent with previous studies of RCM1 (19). The RCM1-encapsulated nanoparticles reduce tumor burden, proliferation, mitosis, and angiogenesis in a dose-dependent manner.

While both VCR and RCM1-encapsulated nanoparticles were able to reduce tumor burden in a dose-dependent manner, we selected IC_50_ concentrations based off of our dose response curves to use in combination *in vivo*. Impressively, while neither IC_50_ concentration of VCR nor RCM1 could reduce tumor burden, the combination therapy with the same doses reduced tumor burden. Consistent with our *in vitro* findings, the combination therapy synergized to reduce proliferation and mitosis, while also inducing apoptosis. The IC_50_ concentrations of VCR and RCM1 as single agents did not impact angiogenesis, however, the combination therapy was able to reduce angiogenesis. Altogether, our data suggests that the combination therapy is superior to VCR and RCM1 as single agents.

To determine the mechanism of this novel combination therapy, we performed bulk RNA-seq. We identified *Chac1* as a uniquely downregulated gene in the combination treated group compared to singles agents and control. *Chac1* is a gene implicated in regulation of cell death, ER stress and glutathione biosynthesis (65, 66), but the role of *Chac1* in RMS has not been studied. Interestingly, high levels of CHAC1 are associated with a worse prognosis in patients (16, 67). Previous reports have shown that anti-tumor agents can reduce CHAC1 levels in cancer (68, 69). However, there have been no reports on the role of CHAC1 in RMS. Lentiviral knockdown of *Chac1* in murine RMS cells caused a reduction of proliferation, mitosis, and induction of apoptosis. These phenotypes are similar to VCR and RCM1 combination therapy *in vitro* and *in vivo*.

While this study provides insight for a novel combination therapy for RMS, there are limitations. All *in vivo* experiments are conducted within 16 days, due to the untreated tumors rapid growth that required euthanasia per our IACUC protocol. This is a relatively short time and does not allow prolonged examination of the effects of the combination therapy. As a future direction, a survival study could be performed where each animal will be treated until euthanasia criteria are met, including tumor volumes, body weights, etc. Also, the current study has not assessed whether the RCM1/VCR combination therapy could be universally effective for the different subtypes of RMS, including embryonal, alveolar, pleomorphic, and spindle cell/sclerosing variants. Since it is well known that all these factors could greatly affect the chemosensitivity of the tumor (70). Additionally, all *in vivo* studies have been conducted using mouse models. While this is a widely accepted condition, it may not recapitulate the possible effects observed in patients. It is unclear whether nanoparticle delivery of RCM1/VCR combination therapy will have beneficial effect in human RMS. The efficacy of RCM1/VCR combination therapy in RMS can only be determined in clinical trials.

## Data availability statement

The data discussed in this publication have been deposited in NCBI's Gene Expression Omnibus (GEO) and are accessible through the GEO Series accession number GSE223149 (https://www.ncbi.nlm.nih.gov/geo/query/acc.cgi?acc=GSE223149).

## Ethics statement

All animal studies were approved by Cincinnati Children’s Research Foundation Institutional Animal Care and Use Committee and covered under our animal protocol (IACUC2020-0041).

## Author contributions

Conception and Design: JD, TK. Development of methodology: JD, ZD, SS, FB, JG-A. Acquisition of data: JD, ZD, FB, SS, JG-A. Analysis and interpretation of data: JD, ZD, FB, SS, JG-A, TK. Writing, review and/or revision of the manuscript: JD, ZD, FB, SS, JG-A, DS, VK, TK. Study Supervison: TK. All authors contributed to the article and approved the submitted version.

## Supplementary material

Supplementary Figure 1 Bright field images showing that combination treatment with low doses of vincristine and RCM1 is more efficient in decreasing growth of mouse and human rhabdomyosarcoma cells compared to single agents. **(A)** Combination of IC_50_ dose for VCR (2.1nm) and RCM1 (8.7µM) reduced the number of tumor cells compared to IC50 doses of each single agent alone and vehicle (saline + DMSO) control in murine RMS *in vitro*. **(B)** Combination of IC_50_ concentrations for VCR (3.0nm) and RCM1 (3.5µM) reduced the number of tumor cells compared to IC_50_ doses of each single agent alone and vehicle (saline + DMSO) control in human RMS *in vitro*. Scale bar=50µm.

Supplementary Figure 2 Treatment with vincristine reduces tumor burden in a dose-dependent manner in a mouse models of rhabdomyosarcoma. **(A)** Experimental VCR treatment strategy using Rd76-9 as a subcutaneous tumor model. Animals were treated with 0.1mg/kg, 0.2mg/kg or 0.4mg/kg VCR. **(B)** VCR reduced tumor volume compared to vehicle (saline) control. Tumor volume measurements are shown at different time-points (top) and representative tumor images per group (bottom). **(C)** The dose-response curve, showing tumor volumes at day 16 after treatment with different doses of VCR, was used to determine the IC_50_ concentration of VCR *in vivo* (0.25mg/kg). **(D)** No differences were found in body weights of mice from each group. **(E)** VCR reduces proliferation (Ki-67, red) and mitosis (**(F)** PH3, green) of tumor cells compared to vehicle control. **(G)** VCR does not reduce tumor-associated angiogenesis (CD31, green) compared to vehicle control. **(H)** VCR does not induce apoptosis (Cleaved-Caspase 3, red) compared vehicle control. 5 random fields per sample were collected to quantify the number of Ki-67^+^, PH3^+^, CD31^+^ and Cleaved-Caspase 3^+^ cells per group. Values are shown as mean ± SD. n=4, *, *P*<0.05; ***, *P*<0.001. Scale bar=50µm.

Supplementary Figure 3 Treatment with RCM1-NP^FA^ reduces tumor growth in a dose-dependent manner in a mouse models of rhabdomyosarcoma. **(A)** The dose-response curve, showing tumor volumes at day 16 after treatment with different doses of RCM1-NPFA, was used to determine the IC50 concentration of RCM1-NP^FA^ *in vivo* (8µg). **(B)** No differences in body weights of mice from each group were found. **(C)** RCM1-NP^FA^ reduced tumor cell proliferation (Ki-67, red) and mitosis (**D** PH3, green) compared to vehicle (empty-NP^FA^) control. **(E)** RCM1-NP^FA^ reduces tumor-associated angiogensis (CD31, green) compared to vehicle control. **(F)** RCM1-NP^FA^ does not induce apoptosis of tumor cells (Cleaved-Caspase 3, red) compared vehicle control. 5 random fields per sample were collected to quantify the number of Ki-67^+^, PH3^+^, CD31^+^ and Cleaved-Caspase 3^+^ cells per group. Values are shown as mean ± SD. n=3-7, **, *P*<0.01; ***, *P*<0.001. Scale bar=50µm.

Supplementary Figure 4 Combination therapy with low doses of VCR and RCM1-NP^FA^ is non-toxic in a mouse model of rhabdomyosarcoma. **(A)** No differences were found in body weights of mice from different groups treated either with single agents or with combination of VCR and RCM1-NP^FA^. **(B–D)** No differences were found between groups across different liver enzymes in serum. Saline + empty-NP^FA^ was used as a vehicle control. Values are shown as mean ± SD. n=5-6 per group, *P*>0.05.
