# Corrigendum: Improving anti-tumor efficacy of low-dose Vincristine in rhabdomyosarcoma *via* the combination therapy with FOXM1 inhibitor RCM1

**DOI:** 10.3389/fonc.2023.1163510

**Published:** 2023-04-13

**Authors:** Johnny Donovan, Zicheng Deng, Fenghua Bian, Samriddhi Shukla, Jose Gomez-Arroyo, Donglu Shi, Vladimir V. Kalinichenko, Tanya V. Kalin

**Affiliations:** ^1^ Division of Pulmonary Biology, Cincinnati Children’s Hospital Medical Center, Cincinnati, OH, United States; ^2^ The Materials Science and Engineering Program, College of Engineering and Applied Science, University of Cincinnati, Cincinnati, OH, United States; ^3^ Center for Lung Regenerative Medicine, Cincinnati Children’s Hospital Medical Center, Cincinnati, OH, United States; ^4^ Division of Pulmonary and Critical Care and Sleep Medicine, Department of Internal Medicine, University of Cincinnati, Cincinnati, OH, United States

**Keywords:** FOXM1 inhibitor, combination therapy, rhabdomyosarcoma, nanoparticles, animal models

**Error in Figure/Table**

In the published article, there was an error in **Figure 3**, panel J as published. We inadvertently used the incorrect image for the RCM1-NPFA IVIS image on Day 9 in panel 3J. The corrected **Figure 3** and its caption appear below. 

**Figure 3:**
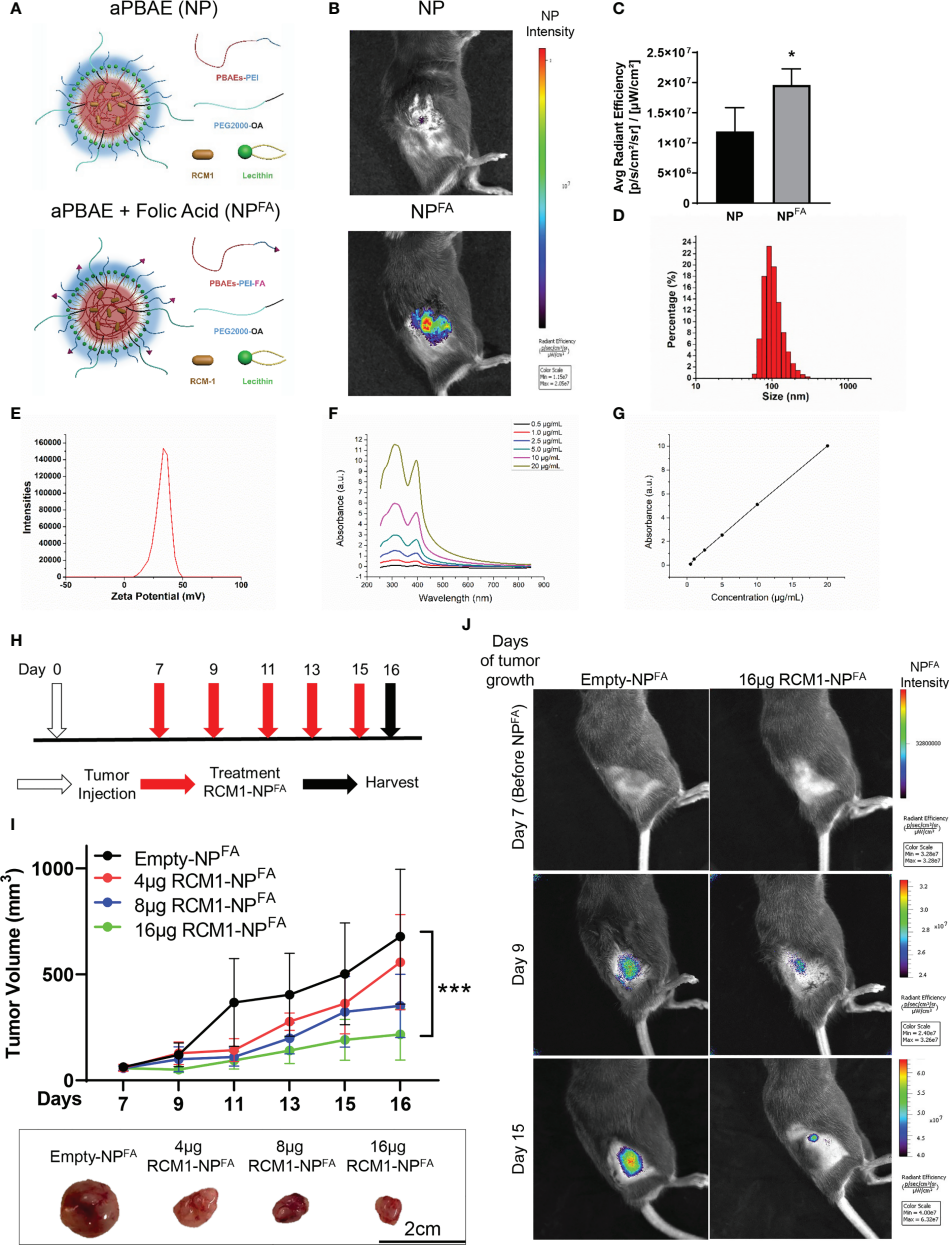
Generation of nanoparticle to deliver RCM1 to tumors. **(A)**, Graphic of amphiphilic poly-beta amino ester (aPBAE) nanoparticles without folic acid (NP, top) and with folic acid (NP^FA^, bottom). **(B)**, Highly efficient delivery of NP^FA^, compared to NP into the tumors is shown using IVIS imaging. Mice bearing Rd76-9 subcutaneous RMS tumors were injected with NP or NP^FA^ labeled with DyLight 800. NP^FA^ are present in the tumor 48 hours after i.v. injection. **(C)**, Average radiant efficiency indicating NP^FA^ have a higher intensity compared to NP. **(D)**, The sizes of NP^FA^ were measured and the hydrodynamic average diameter of NP^FA^ is 160.67nm. **(E)**, The surface charge of NP^FA^ is 38.13mV. **(F)**, The UV/Vis spectrum for RCM1 at increasing concentrations in DMSO determined RCM1 has an absorbance peak at 310nm and 395nm. **(G)**, Data from the UV/VIS spectra was used to generate a standard concentration curve that was used to determine RCM1 concentration in the nanoparticles. Adjusted R^2 =^ 0.99944. **(H)**, Schematic diagram showing treatment strategy of tumor bearing mice. Rhabdomyosarcoma Rd76-9 cells were inoculated subcutaneously. Animals were treated with 4µg, 8µg, or 16µg RCM1- NP^FA^. **(I)**, Treatment with RCM1-NP^FA^ reduced tumor burden in a dose-dependent manner in animals. Mice were treated with 4µg, 8µg, or 16µg of RCM1-NP^FA^ or with empty-NP^FA^. Tumor volumes were measured at different time points compared to empty-NP^FA^ (top panel). Representative tumors per group are shown (bottom panel). Values are shown as mean ± SD. n=3-7, *P<0.05; ***P<0.001. **(J)**, Presence of Empty-NP^FA^ and RCM1-NP^FA^ nanoparticles in the tumors are shown at days 9 and 15 after treatment. Nanoparticles were labeled with DyLight 800 and visualized using IVIS.

The authors apologize for this error and state that this does not change the scientific conclusions of the article in any way. The original article has been updated.

