# Supplementary figures and images for: Improving anti-tumor efficacy of low-dose Vincristine in rhabdomyosarcoma via the combination therapy with FOXM1 inhibitor RCM1
Source: Front Oncol. 2023 Feb 2;13:1112859. doi: 10.3389/fonc.2023.1112859 (PMC9933126; doi:10.3389/fonc.2023.1112859)

**A**

Mouse  
Rd76-9

Vehicle

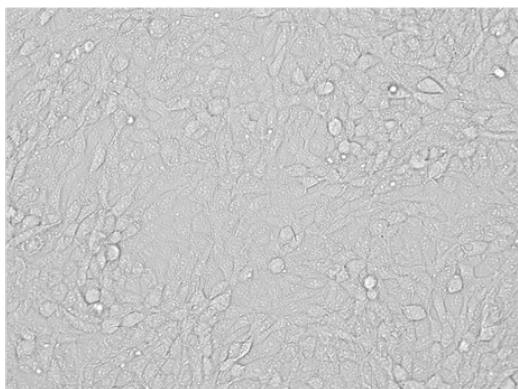

VCR

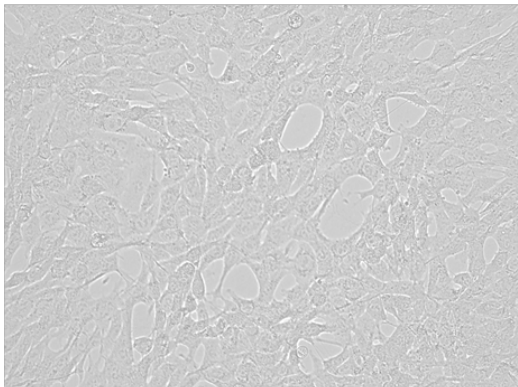

RCM1

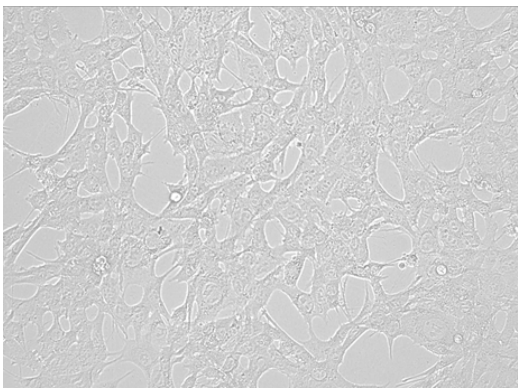

VCR + RCM1

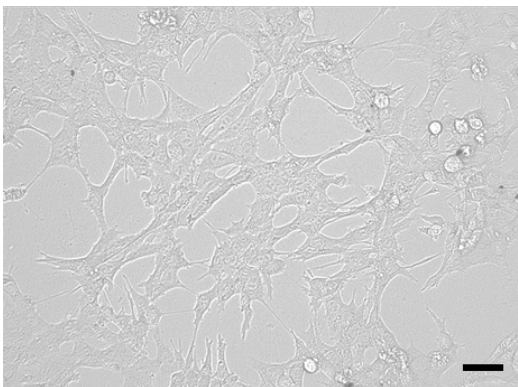

**B**

Human  
RD

Vehicle

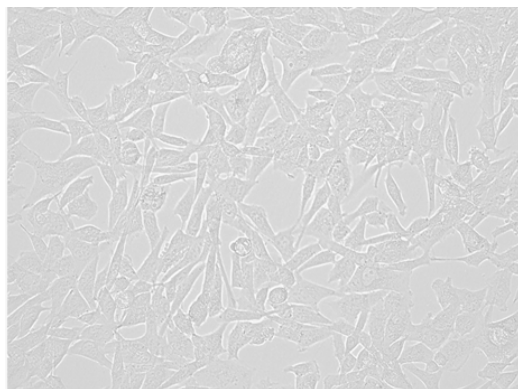

VCR

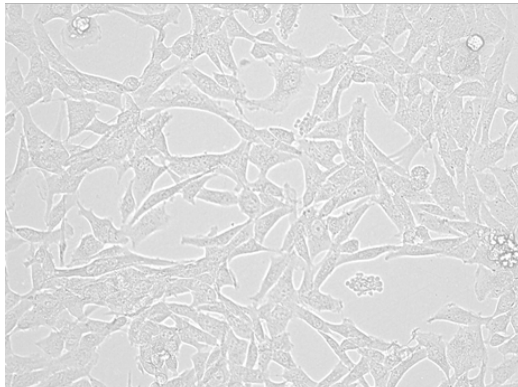

RCM1

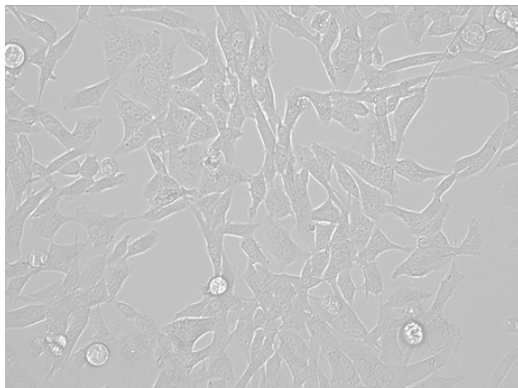

VCR + RCM1

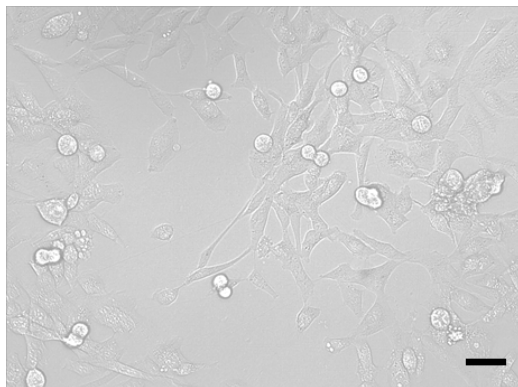

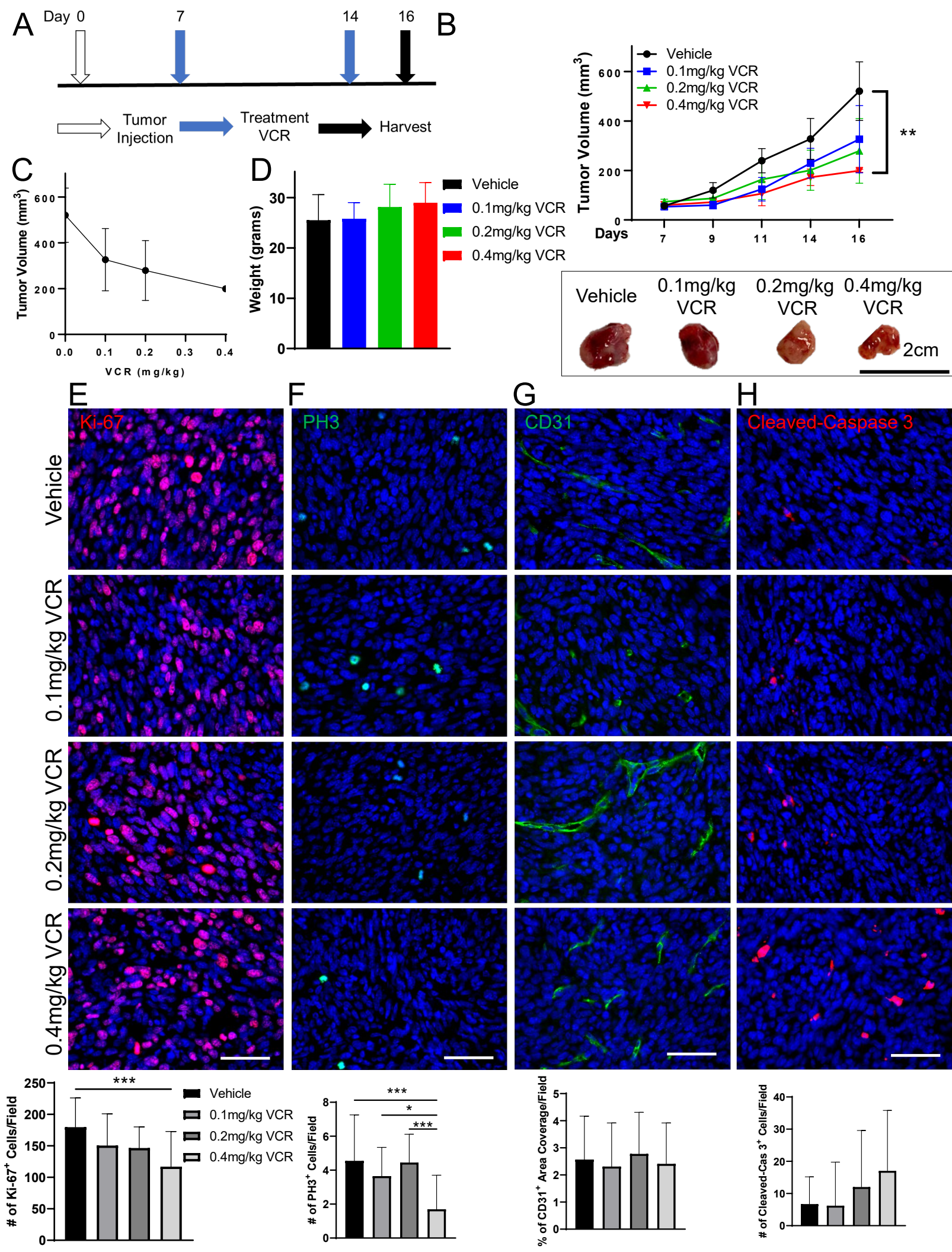

A

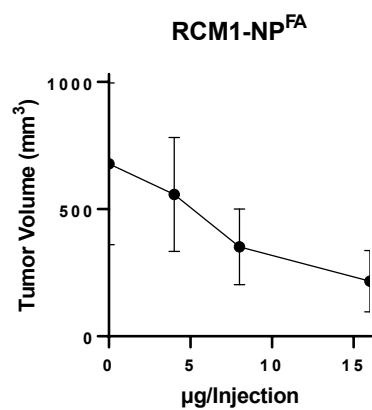

B

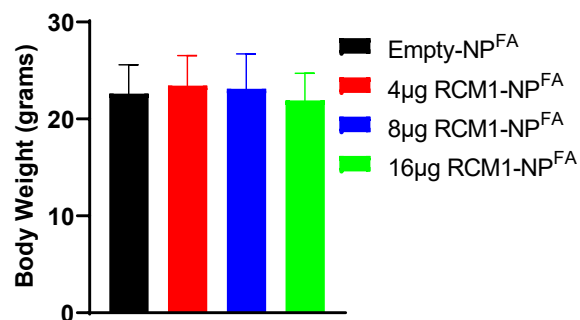

C

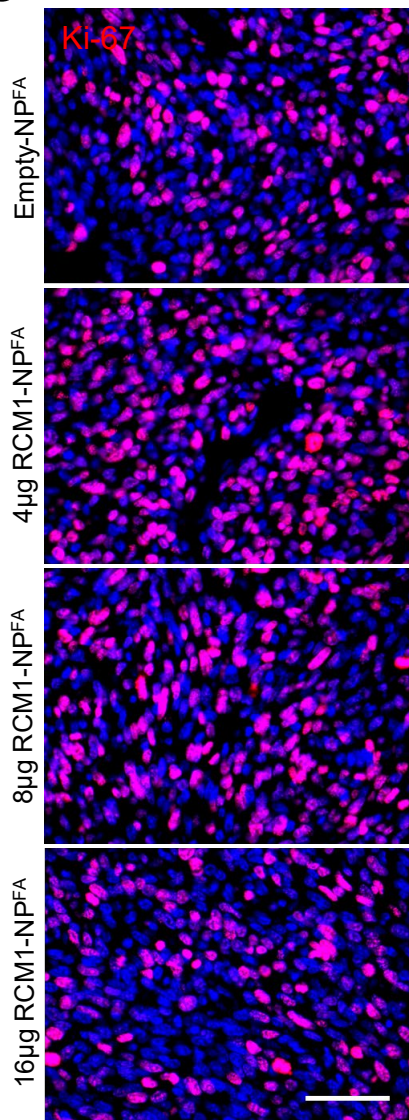

D

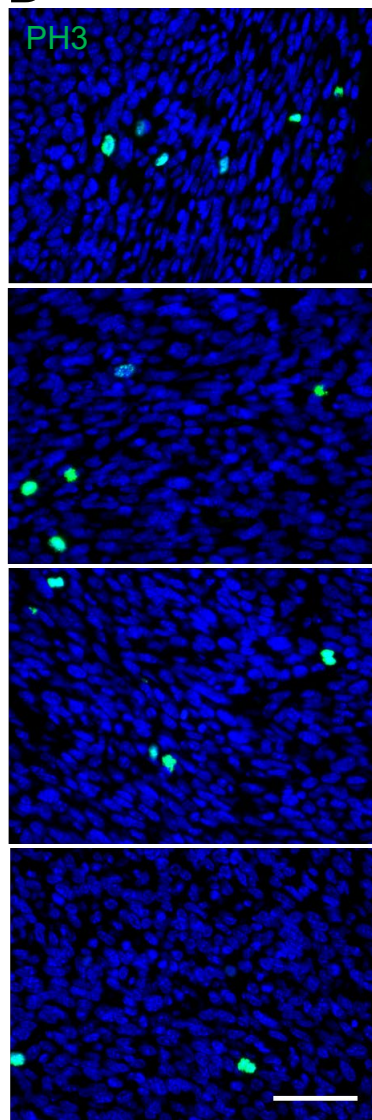

E

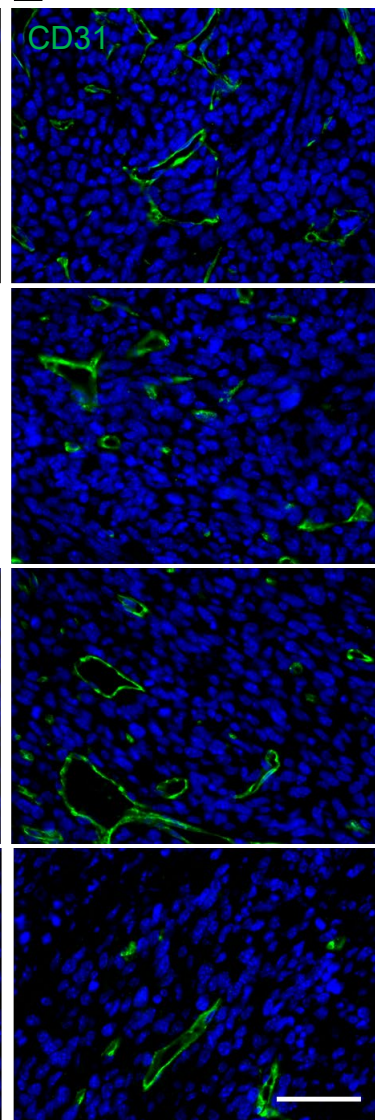

F

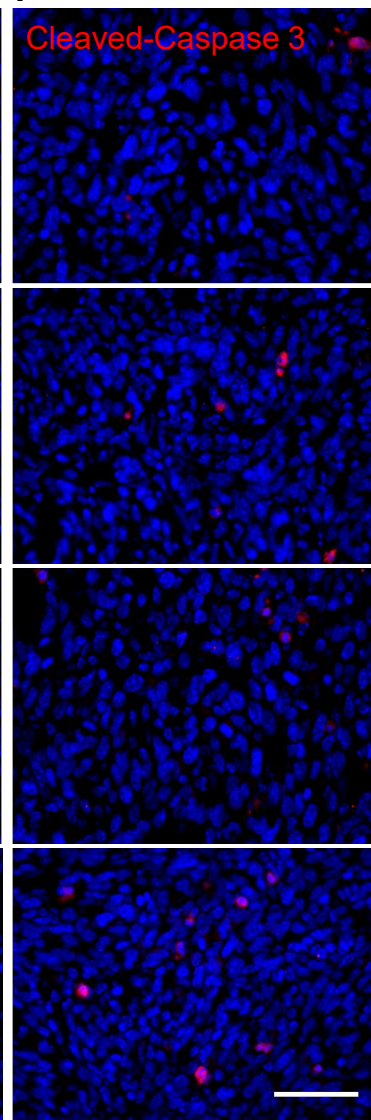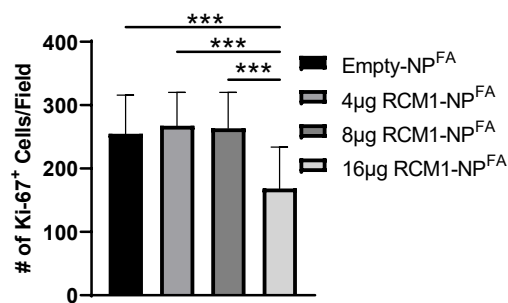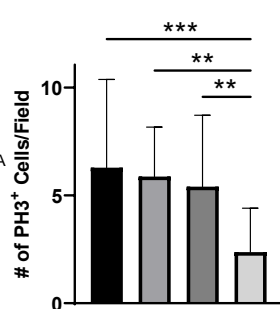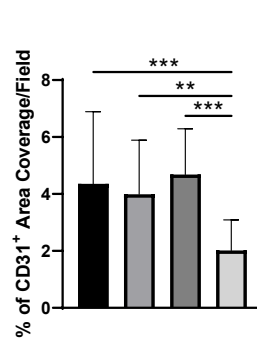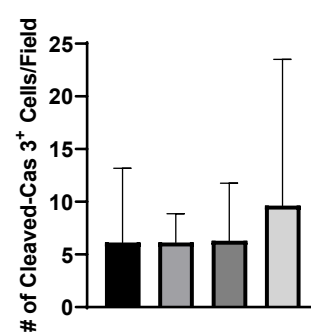

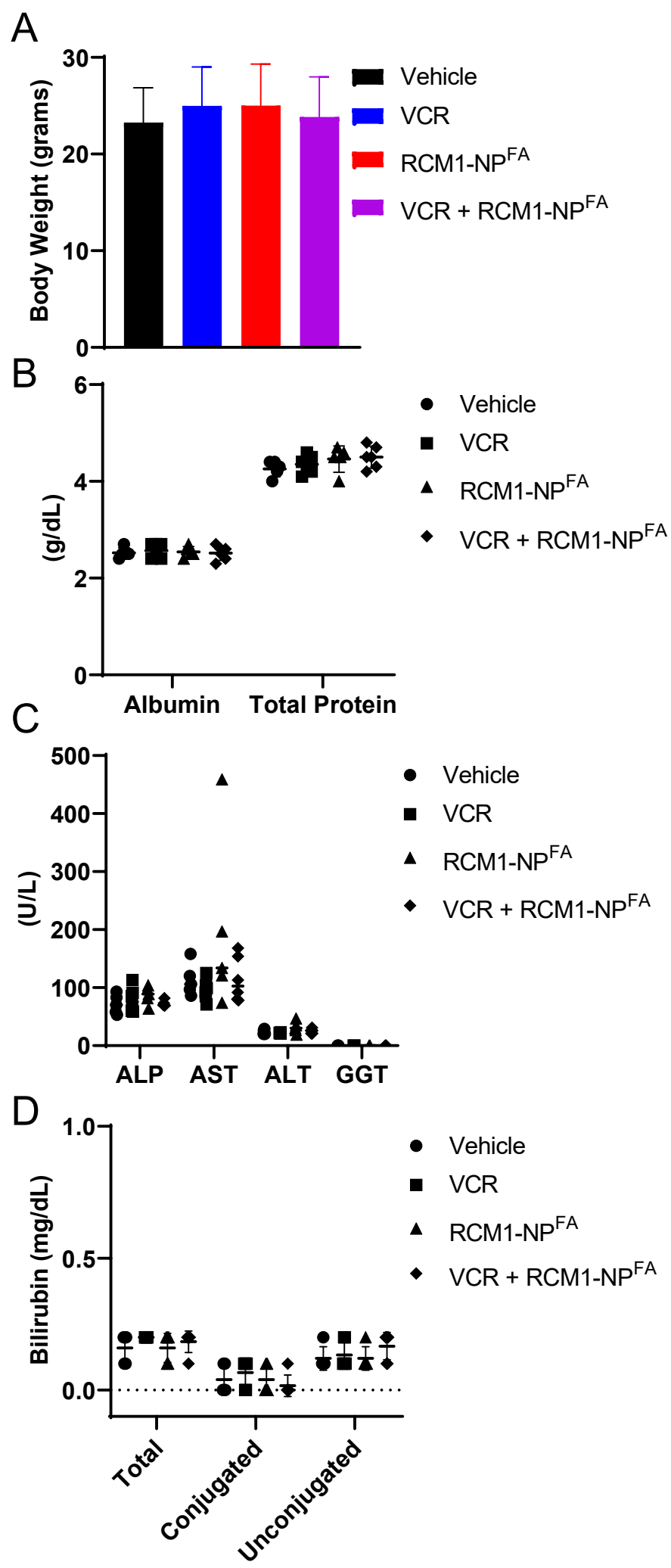

Supplement: Supplementary Figure 1 — Bright field images showing that combination treatment with low doses of vincristine and RCM1 is more efficient in decreasing growth of mouse and human rhabdomyosarcoma cells compared to single agents. (A) Combination of IC50 dose for VCR (2.1nm) and RCM1 (8.7µM) reduced the number of tumor cells compared to IC50 doses of each single agent alone and vehicle (saline + DMSO) control in murine RMS in vitro. (B) Combination of IC50 concentrations for VCR (3.0nm) and RCM1 (3.5µM) reduced the number of tumor cells compared to IC50 doses of each single agent alone and vehicle (saline + DMSO) control in human RMS in vitro. Scale bar=50µm. [file DataSheet_1.pdf]
